# Interventions to Increase Patient Safety in Long-Term Care Facilities—Umbrella Review

**DOI:** 10.3390/ijerph192215354

**Published:** 2022-11-21

**Authors:** Jakub Świtalski, Katarzyna Wnuk, Tomasz Tatara, Wojciech Miazga, Ewa Wiśniewska, Tomasz Banaś, Olga Partyka, Katarzyna Karakiewicz-Krawczyk, Justyna Jurczak, Mateusz Kaczmarski, Grażyna Dykowska, Aleksandra Czerw, Elżbieta Cipora

**Affiliations:** 1 Department of Health Economics and Medical Law, Faculty of Health Sciences, Medical University of Warsaw, 01-445 Warsaw, Poland; 2 Department of Health Policy Programs, Department of Health Technology Assessment, Agency for Health Technology Assessment and Tariff System, 00-032 Warsaw, Poland; 3 Department of Public Health, Faculty of Health Sciences, Medical University of Warsaw, 02-091 Warsaw, Poland; 4 Department of Gynecology and Obstetrics, Jagiellonian University Medical College, 31-501 Cracow, Poland; 5 Department of Radiotherapy, Maria Sklodowska-Curie Institute-Oncology Centre, 31-115 Cracow, Poland; 6 Department of Economic and System Analyses, National Institute of Public Health NIH—National Research Institute, 00-791 Warsaw, Poland; 7 Department of Clinical Nursing, Pomeranian Medical University in Szczecin, 71-210 Szczecin, Poland; 8 Department of Social Medicine and Public Health, Pomeranian Medical University in Szczecin, 71-210 Szczecin, Poland; 9 Medical Institute, Jan Grodek State University in Sanok, 38-500 Sanok, Poland

**Keywords:** patient safety, long-term care, elderly people

## Abstract

Introduction: Patient safety in long-term care is becoming an increasingly popular subject in the scientific literature. Organizational problems such as shortages of medical staff, insufficient numbers of facilities or underfunding increase the risk of adverse events, and aging populations in many countries suggests that these problems will become more and more serious with each passing year. The objective of the study is to identify interventions that can contribute to increasing patient safety in long-term care facilities. Method: A systematic review of secondary studies was conducted in accordance with the Cochrane Collaboration guidelines. Searches were conducted in Medline (via PubMed), Embase (via OVID) and Cochrane Library. The quality of the included studies was assessed using AMSTAR2. Results: Ultimately, 10 studies were included in the analysis. They concerned three main areas: promoting safety culture, reducing the level of occupational stress and burnout, and increasing the safety of medication use. Promising methods that have an impact on increasing patient safety include: preventing occupational burnout of medical staff, e.g., by using mindfulness-based interventions; preventing incidents resulting from improper administration of medications, e.g., by using structured methods of patient transfer; and the use of information technology that is more effective than the classic (paper) method or preventing nosocomial infections, e.g., through programs to improve the quality of care in institutions and the implementation of an effective infection control system. Conclusions: Taking into account the scientific evidence found and the guidelines of institutions dealing with patient safety, it is necessary for each long-term care facility to individually implement interventions aimed at continuous improvement of the quality of care and patient safety culture at the level of medical staff and management staff.

## 1. Introduction

In recent years, long-term care has become the subject of analyses carried out by the largest entities acting both directly and indirectly to ensure the protection of human health in the world. Projects are carried out by the World Health Organization (WHO), the Organization for Economic Cooperation and Development (OECD), Eurostat, and the National Institute on Aging (NIA) [1,2,3,4]. These interventions are taken due to the noticeable deficits in the organizational scope of long-term care. Shortages of medical staff, insufficient numbers of facilities or underfunding increase the risk of adverse events, and aging populations in many countries allow us to predict that these problems will become more and more serious with each passing year [5,6,7].

Regardless of the country, the specificity of long-term care benefits is similar. Treatment of chronic diseases, rehabilitation, palliative care and preventive interventions are most often undertaken within its framework [8]. These benefits are provided at home or within resident facilities [4]. In the case of the second form of assistance, just like in any entity operating within the health care system, apart from the positive effects of the actions taken, there are also negative ones, i.e., the occurrence of adverse events and medical errors. The results of the conducted research show that about half of these events are related to the care provided. The formation of pressure ulcers, other wounds and patients’ falls, and consequently injuries, constitute a serious problem that significantly worsens the health of already ill patients. Emerging infections and undesirable effects of medications are also problematic. The most important information, however, is that approximately 70% of these problems can be avoided by taking actions aimed at increasing patient safety and safety culture [9]. These types of actions play a major role in reducing patients’ pain and suffering. At the same time, due to the costs resulting from adverse events and medical errors, from the perspective of decision-makers and payers, it is important to implement all possible actions limiting the negative effects of providing care [10,11]. The development of patient safety prevents the generation of additional costs for the operation of facilities. The moral aspect should also be mentioned, i.e., the obligation to provide care for the elderly and to ensure that they can age in a dignified way and in a safe place, which is also pointed out by WHO [8].

Medical staff plays a key role in patient care. Therefore, a comprehensive assessment of the safety culture of healthcare institutions should include the study of the impact of the human factor on patient safety. Due to the nature of the tasks performed, the work of nurses, doctors, physiotherapists and other medical professionals is subject to a high risk of error. Therefore, it is necessary to optimize the procedures and behavior of medical staff so that the number of adverse events and errors is kept to a minimum. The cooperation of therapeutic teams and appropriate communication within them may contribute to the improvement of the patients’ situation. The management of institutions also plays an important role in the process of creating a climate of safety [12]. Research indicates that improved cooperation between healthcare professionals can significantly affect patient safety by reducing complications and mortality [13]. A study published in 2016 examining the impact of the well-being/satisfaction with work of medical staff on patient safety indicated that a low level of well-being and moderate and high rates of occupational burnout of medical staff result in a lower level of patient safety (expressed, among others, in the growth of the occurrence of medical errors) [14]. However, it should highlighted that most of the research was carried out in facilities other than those providing long-term care. The objective of this study is to identify interventions that can contribute to increasing patient safety in long-term care facilities.

An umbrella review was conducted to identify studies at the highest level of the hierarchy of scientific evidence. The above enabled a collective analysis of the available scientific evidence in the discussed area. The research conducted so far has focused on various areas, interventions and sub-populations. Conducting a review of secondary research and its analysis allows for the organization and synthetic presentation of data so as to facilitate access to collective information for people interested in the subject of patient safety in long-term care facilities, including representatives of medical professions and decision makers.

## 2. Materials and Methods

The study takes into account secondary scientific evidence published between 2010 and 2022. The originally performed search covered the period from 1 January 2010 to 14 March 2021. Due to the fact that relevant publications could have been published after this period, the search was updated covering the period from 14 March 2021 to 24 May 2022. The above made it possible to find the most up-to-date and reliable data. The search was performed in Medline (via PubMed), Embase (via OVID) and Cochrane Library, in accordance with the previously prepared strategy (supplementary material).

The systematic review was carried out according to the Cochrane Collaboration guidelines [15]. The studies were searched for on the basis of a detailed protocol developed prior to the commencement of work. It took into account the criteria for including studies in the review, the search strategy, the method of selecting studies, and the planned methodology for conducting data analysis and synthesis. The inclusion criteria for this analysis are presented in Table 1. Taking into account the broad scope of the study, it was decided not to limit the search to specific comparators.

At all stages of the systematic review, the selection of studies was performed by two people working independently (J.Ś., K.W.). Inconsistencies were resolved by consensus with the participation of a third independent analyst (T.T.).

**Figure 1:**
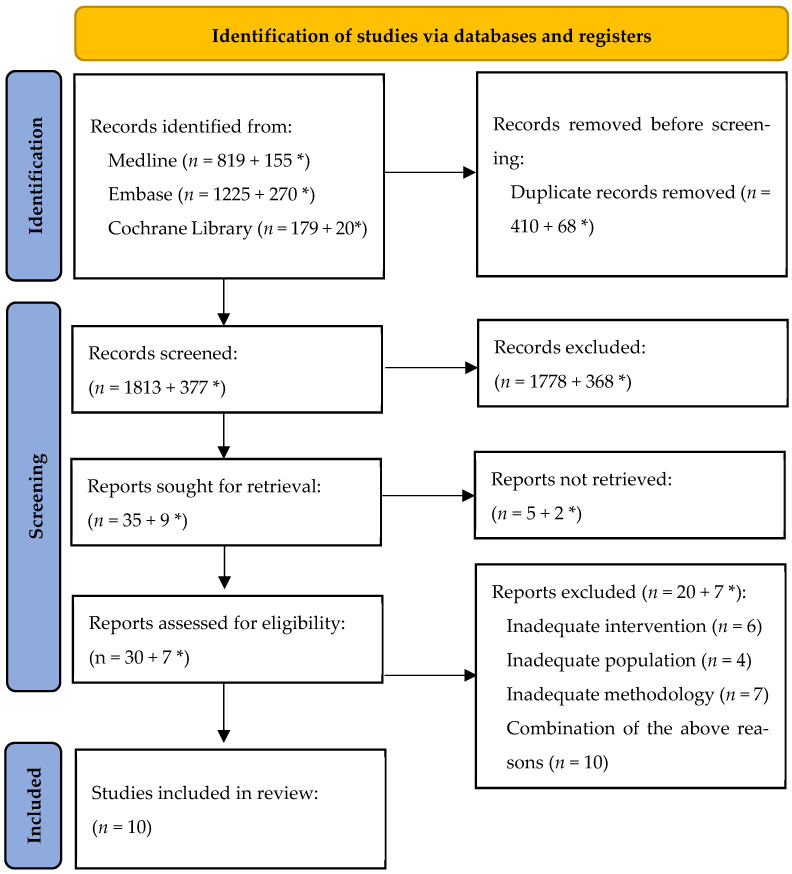
PRISMA flow diagram. * studies found as a result of a search update conducted for the period from 14 March 2021 to 24 May 2022.

As a result of the first systematic search, 2223 secondary studies were found. After removing duplicates (using the Reference Manager), 1813 secondary scientific studies were included in the abstract analysis. The analytical process then identified 35 studies that potentially met the criteria for inclusion in the review. The most common reasons for excluding studies from the analysis were issues related to the methodology (lack of correct description of the material and method, incorrect synthesis of the review results) and the lack of a sufficient description of the interventions used. After verifying the full texts, 10 publications were included in the review. As a result of the supplementary search, 445 studies were found, none of which finally met the criteria for inclusion in the analysis. The study selection steps are presented in Figure 1. The list of included and excluded publications is in the Supplementary Material.

Due to the limited amount of scientific evidence relating directly to the situation in long-term care facilities, the analysis also included the results of studies from other facilities (e.g., primary care facilities, hospital wards), from which the interventions applied can be extrapolated to facilities caring for the elderly.

The quality of secondary studies included in the analysis was assessed by verifying the key domains of the tool for critical evaluation of systematic reviews—AMSTAR2 [16]. The applied tool allows for the identification of publications (including randomized and non-randomized studies) of the highest quality. It should be emphasized, however, that the tool is not intended to include or exclude publications from the research review, but is only a guide in determining the potential value of the results obtained. A publication with positive answers to all questions had the highest grade (the criterion was allowed to be partially met). On the other hand, one deficiency (answer “no”) in the critical domain results in lowering the evaluation of the systematic review to the value “low”. Two or more deficiencies lower the grade to “critically low”. The quality assessment was performed by two independently working analysts (W.M. and E.W.). Inconsistencies were resolved by consensus with the participation of a third independent analyst (J.Ś.). Detailed results of the quality analysis and the risk of error are included in the supplementary material.

## 3. Results

The following scientific evidence met the inclusion criteria for a systematic review involving the analysis of interventions to improve patient safety (*n* = 10; Kruse 2021 [17], Bukoh 2020 [18], McCarthy 2018 [19], Burton 2017 [20], Alldred 2016 [21], Busireddy 2016 [22], Hill 2016 [23], Snowdon 2016 [24], Marasinghe 2015 [25], Weaver 2013 [26]):

- Kruse 2021—a systematic review based on twelve observational studies and two RCTs, assessing the relationship between health information technology and quality improvement in the field of issuing prescriptions in long-term care facilities [17];
- Bukoh 2020—a meta-analysis of nine RCTs and quasi-experimental studies, which assessed the effectiveness of structured handover interventions between nurses and their impact on improving the quality and safety of patient care [18];
- McCarthy 2018—a systematic review based on six uncontrolled pre/post intervention studies, analysing the impact of filling electronic documentation by nurses on promoting/improving the quality of care and/or patient safety in hospital wards [19];
- Burton 2017—a meta-analysis of seven pre/post studies (with or without a control group) and two RCTs, assessing the effectiveness of mindfulness-based interventions in reducing stress among medical staff [20];
- Alldred 2016—a systematic review based on 12 RCTs that assessed the effect of interventions to optimize the medication delivery process for elderly people living in nursing homes [21];
- Busireddy 2016—a meta-analysis of six RCTs and thirteen cohort studies, assessing the effectiveness of mindfulness-based interventions in reducing stress among medical staff [22];
- Hill 2016—a systematic review based on nine publications (RCT, non-RCT, pre/post studies and process evaluation studies), which analyzed the impact of psychological interventions on improving the well-being of medical staff in palliative care facilities [23];
- Snowdon 2016—a meta-analysis of 32 cohort studies (prospective and retrospective) and pre/post studies, assessing the effectiveness of clinical supervision among medical staff in improving patient safety [24];
- Marasinghe 2015—a systematic review based on five RCTs and two cohort studies, which analyzed the impact of computerized clinical decision support systems on the safety of medication administration in long-term care facilities [25];
- Weaver 2013—a systematic review based on 33 publications (27 pre/post studies, 3 studies using time-series analysis and 3 RCTs), which identified and analyzed the effectiveness of interventions used to promote safety culture or climate in hospital wards [26].

The results of the studies found are presented below.

### 3.1. Promoting Safety Culture in A Facility

Research on promoting safety culture in a facility concerned, among others, methods of transferring care between nursing teams or the manner of keeping medical records (Bukoh 2020 [18], McCarthy 2018 [19], Snowdon 2016 [24], Weaver 2013 [26]).

According to the results of the Bukoh 2020 [18] meta-analysis, structured methods of patient transfer (SBAR—situation, background, assessment, recommendation; ICCCO—identification of the patient and clinical risks, clinical history/presentation, clinical status, care plan and outcomes/goals of care; PCH—patient-centred handovers; NHF—standardized nursing handoff form) can improve patient safety by reducing errors related to medication administration—SMD = −0.07 [95%CI: −0.13; −0.01, *p* = 0,02]. The obtained effect was, however, small, and the remaining results concerning the influence of the applied intervention on the reduction of complications after treatment (SMD = −0.15 [95% CI: −0.32; 0.01, *p* = 0.07]), the occurrence of adverse events (SMD = −0.13 [ 95% CI: −0.26; 0.01, *p* = 0.06]), and errors in transferring nursing care (SMD = −0.47 [95% CI: −1.04; 0.09, *p* = 0.1]) were statistically insignificant [18].

On the other hand, the McCarthy 2018 [19] study analysing the impact of electronic filing by nurses on promoting/improving the quality of care and/or patient safety showed that there is a possibility of reducing the time needed to complete records within a short follow-up period—3 months with the use of an electronic system only (from 138.5 h to 55.8 h a week). It seems that electronic systems for keeping medical records are more effective than the classic (paper) system; however, the conclusions from the study are limited due to the small number of publications analysing this issue [19].

The Snowdon 2016 [24] meta-analysis examined the impact of introducing clinical supervision for medical staff on patient safety. The authors of the study indicate that for the purposes of the described publication, clinical supervision is defined as presenting clinical guidelines for medical staff by more experienced employees. The purpose of supervision may be, for example, to facilitate work, emotional support, and systematization of knowledge. However, the main assumption of clinical supervision is to achieve high-quality patient care. In accordance with the results of the meta-analysis of 14 studies (*n* = 20.474), there was low-quality evidence of a reduction in the risk of death among patients after the intervention compared to clinical supervision with little or no monitoring—RR = 0.76 [95% CI: 0.60; 0.95, I^2^ = 76%]. Due to the high level of heterogeneity, a subgroup analysis was performed with nine studies (*n* = 6.484) showing moderate-quality evidence of a reduction in the risk of death among patients after clinical (versus no) supervision following surgery—RR = 0.68 [95% CI 0.50; 0.93, I^2^ = 33%]. A meta-analysis of 23 studies (*n* = 104.625) showed low-quality evidence of a reduction in the risk of treatment-related complications after the intervention as compared to low or no intervention—RR = 0.69 [95% CI: 0.53; 0.89, I^2^ = 76%]. Moreover, due to the high level of heterogeneity, the authors conducted a subgroup analysis based on three studies (*n* = 23.609) that showed moderate-quality evidence that the risk of treatment-related complications was reduced after clinical (versus no) supervision after invasive treatment and surgery—RR = 0.33 [95% CI 0.24; 0.46, I^2^ = 0%]. However, after analysing 16 studies (*n* = 66.447), low-quality evidence was obtained, indicating no statistically significant reduction in the risk of treatment-related complications after direct clinical supervision during surgery (compared to without it)—RR = 0.85 [95% CI 0.67; 1.07, I^2^ = 61%]. Considering reoperations as the endpoint, the meta-analysis of nine studies (*n* = 10.699) showed no statistically significant differences between the reoperation rate in the group of patients under clinical supervision and those not undergoing this intervention—RR = 1.16 [95% CI: 0.92; 1.47, I^2^ = 0%]. However, the authors performed a subgroup analysis of three studies (*n* = 241) that showed moderate-quality evidence of a reduced risk of changing the nature of surgery (e.g., from laparoscopy to open surgery) after clinical supervision (versus no supervision)—RR = 0.39 [95% CI 0.22; 0.69, I^2^ = 0%]. The credibility of the obtained results of meta-analyses is ambiguous due to the high level of heterogeneity, resulting, for example, from different models of supervision and the type of care provided in the analyzed primary studies [24].

In the Weaver 2013 [26] systematic review, 33 studies met the inclusion criteria for the review, of which 21 analyzed patient safety culture using the Safety Attitudes Questionnaire (SAQ), 10 used the Hospital Survey on Patient Safety Culture (HSOPSC), and 2 used the Patient Safety Climate in Healthcare Organizations survey (PSCHO). The vast majority of the analyzed studies showed a statistically significant impact of the intervention on the level of patient safety culture. After using training in communication in therapeutic teams, 16 out of 20 analyzed studies showed a statistically significant improvement in the awareness of the importance of maintaining a high level of safety culture in a facility. There were also reductions in care delays, an increase in structured communication techniques, and a reduction in the number of patient care-related adverse events. One study found a statistically insignificant effect of reducing the cost of care by approximately USD 24, despite extending the patient’s stay in the ward by 0.19 days. In the case of applying comprehensive safety improvement programs, six out of eight studies showed a statistically significant improvement in the awareness of the importance of maintaining a high level of safety culture in a given facility. In addition, two studies included in the review indicated an improvement in the care process, and one study showed a reduction in the incidence of medical errors (0.56 vs. 0.15, *p* < 0.01). However, it should be emphasized that the quality of the studies included in the review was low, so the possibility of drawing conclusions based on them is limited. Moreover, the high level of heterogeneity made it impossible to conduct a meta-analysis [26].

Detailed characteristics and the results of research on promoting safety culture in facilities are presented in Table 2.

### 3.2. Reducing the Level of Occupational Stress and Burnout, as Well as Improving the Well-Being of Medical Staff

Research on occupational stress and burnout, as well as improving the well-being of medical staff, included, among others, mindfulness-based interventions. It was decided to include studies on the discussed issues because of their impact on the quality of care provided and the number of adverse events [27,28,29].

The results of the Burton 2017 [20] meta-analysis indicated that the use of mindfulness-based interventions is effective in minimizing stress (*p* < 0.0002) among medical staff. The results of individual studies indicate a moderate effect of the actions, depending on the duration of the intervention (from 1 day to 10 weeks, depending on the publication). Studies analysing traditional mindfulness-based stress reduction training (intervention duration 4–8 weeks) also showed statistically significant reductions in stress, anxiety and depression. There was also an improvement regarding the sense of occupational burnout and an improvement in well-being. Similar results were obtained for all other analyzed interventions [20].

Busireddy’s 2016 [22] meta-analysis analyzed interventions aimed at reducing occupational burnout. It was found that the introduction of working time limits may positively affect the level of occupational burnout among residents (and other medical professionals). The results of the meta-analysis showed that the reduction in the number of hours (to 80 per week) worked by residents was associated with a statistically significant reduction of emotional exhaustion (OR = 0.59 [95% CI 0.45; 0.79, *p* < 0.001]) and a reduction in the level of occupational burnout (MD = −2.70 [95% CI −3.98; −1.41, *p* < 0.001]). The aggregated results of two studies analysing the impact of the introduction of working time limits on the overall result of occupational burnout showed that there was a lower chance of high occupational burnout scores among residents after the intervention—OR = 0.60 [95% CI 0.37; 0.98, *p* < 0.04] [22].

In turn, the results of the Hill 2016 [23] systematic review indicated that most of the interventions used (e.g., support groups, stress reduction programs, cognitive therapy) had no effect on endpoints such as the level of well-being or occupational burnout. Additionally, none of the studies showed a significant influence of the actions on the analyzed endpoints (except for art therapy and musicotherapy, for which a moderate effect was demonstrated). At the same time, it should be taken into account that the methodological imperfections of the research (e.g., failure to measure the well-being of staff before the intervention) may distort the final results. Moreover, the authors indicate that, on the basis of the available primary studies, it is not possible to draw unambiguous conclusions regarding the effectiveness of psychological interventions in improving the well-being of medical staff employed in palliative care facilities [23].

Table 2 presents detailed characteristics and the results of studies on reducing the level of occupational stress and burnout, as well as improving the well-being of medical staff.

### 3.3. Increasing the Safety of Use of Medications

The last group of studies included in this review was the analysis of interventions aimed at increasing the safety of use of medications.

The Kruse 2021 [17] systematic review assessed the relationship between the use of information technology in health care and increasing the quality of services provided in long-term care facilities. The obtained results of research from Australia, analysing the use of electronic databases, showed no statistically significant reduction in the time needed for completing medical records by nurses. At the same time, improvements in prescribing adequacy have been demonstrated. Completing the documentation in electronic form has also contributed to increasing its completeness. In turn, studies conducted in Sweden, analysing electronic medication management systems, showed a reduction in the level of stress among medical staff related to the risk of adverse events. Additionally, these types of tools have improved the attitude to conducting administrative activities. Other studies included in the review showed a positive effect of the use of information technology on the quality of information provided during staff shifts [17].

The Alldred 2016 [21] systematic review assessed the effect of interventions to optimize the medication administration process for elderly people living in nursing homes. The research found in the review identified problems with medication administration and prescribing. However, only five RCTs using verification of medication prescription by pharmacists and interdisciplinary discussion meetings of medical staff indicated a benefit from the use of strategies aimed at improving the correctness of medication use. According to the results of the review, a pharmacist’s coordination of medication administration (RR = 1.05 [95% CI 0.66; 1.68]) and the decision support system (aRR = 1.06 [95% CI 0.92; 1.23]) did not statistically significantly affect the occurrence of adverse medication reactions. In the case of the primary endpoint of hospitalization, it was shown that coordinating medication administration by a pharmacist, combined with informing medical staff about medications taken by patients, statistically significantly reduces the number of hospitalizations (RR = 0.38 [95% CI 0.15; 0.99]) in the case of people who were alive at the time of the study. In the analysis of patients who died during the study period, no statistically significant relationship was found—RR = 0.58 [95% CI 0.28; 1.21]. However, in one of the studies it was found that the education of nursing staff reduced the number of days spent in hospital—aRR = 0.60 [95% CI 0.49; 0.75]. On the other hand, in other publications it was stated that both the pharmacist’s verification of medication use (RR = 0.75 [95% CI 0.52; 1.07]) and the education of nurses with interdisciplinary discussion meetings (RR = 1.02 [95% CI 0.83; 1.26]) do not statistically significantly affect the number of hospitalizations. In the case of the next primary endpoint, the pharmacist’s verification of medication use (RR = 1.06 [95% CI 0.70; 1.64]) and nurse education, along with interdisciplinary discussion meetings (RR = 1.11 [95% CI 0.76; 1.61]), also did not statistically significantly affect reducing the risk of death among patients. The authors of the review emphasize that no meta-analysis was performed due to the high level of heterogeneity of the studies [21].

The Marasinghe 2015 [25] systematic review analyzed computerized clinical decision support systems. The results of the individual studies included in the review varied. One study did not show any effect of interventions on the number of adverse events. On the other hand, another study did not show any influence of the undertaken actions on the adequacy of prescribing medications. Most of the studies, however, focused on the detection of elements warning against medication misuse. In one of the studies, computerized decision support systems detected 9414 situations that could have a negative impact on the adequacy of medication administration (a total of 47,977 medication orders were registered). Prescribers receiving alerts from the system were more likely to make optimal treatment decisions—RR = 1.11 [95% CI 1.00; 1.22]. The authors of the review emphasize that such tools may be helpful in increasing the safety of medications, even though they were not often used in long-term care facilities [25].

Detailed characteristics and the results of studies on increasing the safety of medication use are presented in Table 2.

## 4. Discussion

Based on the results of the studies found in the systematic review, an analysis of interventions aimed at increasing patient safety in long-term care facilities was performed.

Among the secondary evidence found in the course of the systematic review, studies were identified on interventions aimed at: promoting safety culture in a given facility (Bukoh 2020 [18], McCarthy 2018 [19], Snowdon 2016 [24], Weaver 2013 [26]); reducing the level of occupational stress and burnout, as well as improving the well-being of medical staff (Burton 2017 [20], Busireddy 2016 [22], Hill 2016 [23]), and increasing the safety of medication use (Kruse 2021 [17], Alldred 2016 [21], Marasinghe 2015 [25]).

Research on promoting safety culture in a facility concerned, inter alia, methods of transferring care between nursing teams and the manner of keeping medical records. According to the results of the Bukoh 2020 meta-analysis, structured methods of patient transfer can improve patient safety by reducing the number of drug administration errors. The obtained effect was, however, small, and the remaining results of the analyzed parameters (the impact of structured methods of patient transfer on the reduction of complications after treatment, occurrence of adverse events, errors in transferring nursing care) were statistically insignificant [18]. The McCarthy 2018 study analyzing the impact of electronic filing of medical records by nurses on promoting/improving the quality of care and/or patient safety showed that it is possible to reduce the time needed to fill medical records in a short follow-up period—3 months after the introduction of an electronic system only (from 138.5 h to 55.8 h a week). It seems that electronic systems for keeping medical records are more effective than the classic (paper) ones, while the conclusions of the study are limited due to the small number of publications analyzing this issue [19].

Research on stress and professional burnout, as well as improving the well-being of medical staff included, inter alia, mindfulness-based interventions. The results of the Burton 2017 meta-analysis indicated that the use of the above-mentioned interventions is effective in minimizing stress and other parameters related to well-being among healthcare professionals. It is not only traditional mindfulness-based stress reduction training that is effective, but also other similar interventions [20]. On the other hand, the results of the Hill 2016 systematic review indicated that, on the basis of the available primary research, it is not possible to draw unambiguous conclusions regarding the effectiveness of psychological interventions in improving the well-being of medical staff employed in palliative care facilities [23]. The Busireddy 2016 meta-analysis analyzed interventions to reduce professional burnout. It was found that the introduction of working time limits may have a positive impact on the level of occupational burnout among residents (the conclusions can, however, be treated as universal in the context of representatives of other medical professions) [22].

The last group of evidence included studies analyzing interventions aimed at increasing the safety of using drugs. In the Kruse 2021 systematic review, assessing the correlation between the use of health information technology to increase the quality of services provided in long-term care facilities, a positive impact of this type of intervention on the reduction of the number of adverse events related to drug administration was noted. However, they did not reduce the time-consuming process of filling medical records by nurses [17]. In the Marasinghe 2015 study, electronic clinical decision support systems were analyzed. They were not often used in long-term care facilities, but the results of a systematic review showed that such tools may be helpful in increasing the safety of drug administration [25]. The Alldred 2016 systematic review assessed the effect of interventions aimed at optimizing the drug administration process for elderly people living in nursing homes. Only five RCTs, which used drug prescription verification by pharmacists and interdisciplinary discussion meetings of medical staff, indicated a benefit from the use of strategies aimed at improving the correct drug administration. However, no evidence was found for the impact of this type of intervention on the incidence of adverse drug reactions, number of hospitalizations, mortality or quality of life of patients [21].

For the purposes of discussion, the current guidelines for the use of interventions and processes affecting patient safety were reviewed. The most important conclusions from the recommendations relating to improving safety culture in facilities (WHO 2021 [30], TJC 2021 [31]), interventions related to reducing the risk of falls (RNAO 2017 [32], AGS/BGS 2011 [33]) and the prevention of infections in a facility (SHEA/APIC 2008 [34]) are presented below.

The authors of the recommendations found agree that the main conclusion to be drawn from the found documents is the need for continuous improvement of the quality of care provided to patients. Increasing the quality of care will have a direct impact on increasing safety culture. Recommendations as tools to achieve this goal include, for example, keeping a register of adverse events and analysis of root causes; encouraging medical staff to improve their competences; adherence to scientific evidence-based pathways and procedures; having emergency plans in place; paying great attention to communication in therapeutic teams [30,31].

At the same time, the found guidelines emphasize that in the context of the most common events occurring in long-term care facilities, the prevention of falls and infections in the facility, as well as events resulting from medication misadministration, is particularly important.

In facilities, it is recommended to implement interventions in the form of regular assessment of patients’ health, including fall risk tests (involving a history of falls, assessment of gait, balance, muscle strength, vision, and monitoring for cardiologic or neurologic disorders). The interventions recommended to reduce the above-mentioned risks are individual, depending on the patient’s condition and capabilities, physical exercise, and the use of safeguards preventing bedridden patients from falling out of bed. For patients at high risk or suspected vitamin D deficiency, supplementation with an appropriate product is possible [32,33].

In accordance with the recommendations, it is also necessary to actively prevent the occurrence of infections in facilities by implementing an effective control system. It is recommended to monitor patients’ health on an ongoing basis, as well as to promptly intervene when an infection is detected. A person from the medical staff or an in-house infection council should be appointed to perform the above tasks [30,34].

Some of the recommendations found indicate medication misadministration (change of products, wrong dosage, wrong administration) as the most common cause of adverse events, regardless of the place of care. Actions should be taken to reduce this type of incident by training medical staff and ensuring adequate human resources in the facilities [30,31].

There are also other key aspects related to patient safety that should not be forgotten. The available scientific evidence shows that the degree of development of communication skills and teamwork determines the number of committed medical errors, and hence also adverse events [35,36,37,38]. The patient care process is complex and multi-stage. Apart from the elements related to the traditional provision of necessary medical procedures, soft skills of medical staff are also important. Often, the patient’s attitude towards the treatment undertaken depends on the method of providing information. When talking with patients, it should be borne in mind that each person is in a different emotional state and has different experiences with the disease. Therefore, it is very important to establish a communicative relationship with patients, involve them in the process and analyse the feedback signals sent by them [39]. In addition to communication with patients, internal communication between individual members of therapeutic teams plays a very important role. Apart from the form, the way of informing is also important. Providing incomprehensible, ambiguous and chaotic messages constitutes a common problem [35,40]. Standardization of certain fixed elements of the conveyed content plays an important role in the communication processes in therapeutic teams. From a quality management perspective, the way information is presented by all team members should be standardized. It is necessary to pay attention to elements such as the vocabulary used during verbal transfer of information or the correctness and comprehensibility of entries in computer systems (e.g., not using abbreviations that may be illegible to others). A well-functioning and effectively cooperating, multidisciplinary team should create an optimal communication model. Potential shortcomings and problems in this respect should be discussed with all its members. The above observations are confirmed by the Bressan 2019 Systematic Review [36].

Another very important aspect is the implementation of a registry of adverse events, which seems to be crucial for improving patient safety in all types of health care facilities. Looking at the cultural aspects and human nature, reflected in the deep-seated reluctance to admit mistakes, it is also a task that is difficult to implement effectively. Paradoxically, speaking out loud about problems in health care does not mean a weakness of an individual, but wisdom and an attempt to improve the situation. Without a comprehensive reformatting of thinking patterns, it will not be possible to implement an effective system for reporting and recording adverse events. Several scientific publications analyzed aspects related to the willingness and frequency of reporting adverse events by medical staff. The Fung 2012 systematic review examined the enabling factors and barriers related to this issue by nurses. In facilities where safety culture was more developed, adverse events were reported more frequently, as well as would-be events (the type of incidents that did not lead to adverse events). The results for the remaining analyzed parameters (age, experience, level of nurses’ education) were inconclusive. In the context of potential barriers, an important element of fear of consequences was indicated. Administrative problems and an overly complicated, time-consuming reporting process can also be significant factors hampering the development of registers [41]. Very similar results were also obtained in the Vrbnjak 2016 systematic review, which analyzed barriers in reporting events related to medication administration by nursing staff [42].

## 5. Limitations

Only publications in English were included in the review. The studies included in the secondary evidence covered a diverse population in terms of ethnicity and geography. The vast majority of them were observational studies. Moreover, they were characterized by high heterogeneity and different methods of presenting the analyzed data were used. A small number of high-quality primary studies results in the lack of possibility to draw unambiguous and precise conclusions about the effectiveness of the analyzed interventions.

Additionally, in some of the secondary studies included in the analysis, the risk of bias was not assessed or was assessed incorrectly. This causes a significant limitation in drawing conclusions about the effectiveness of interventions undertaken in them.

Another limitation is the small number of studies related to long-term care. For the purposes of this study, it was decided to include studies that also refer to other types of care, which in the described scope can be extrapolated to long-term care in the review.

In addition, it should be borne in mind that the publications found were created in relation to the cultural, economic context and the way the healthcare system of their countries of origin functions. Thus, the effectiveness of the interventions they describe may be different in other health care systems.

## 6. Conclusions

On the basis of the available primary studies and secondary studies, it is not possible to draw unambiguous and precise conclusions regarding the effectiveness of the use of interventions aimed at improving patient safety in long-term care facilities.

However, it seems that the main elements increasing the level of patient safety include:

- prevention of events resulting from medication misadministration—e.g., through structured methods of patient transfer and the use of information technology that is more effective than the classic (paper) one;
- preventing occupational burnout of medical staff—e.g., by using mindfulness-based interventions or introducing working time limits among residents and representatives of other medical professions;
- prevention of falls among patients—e.g., through risk awareness activities, fall risk testing and exercise;
- prophylaxis of infections in facilities—e.g., through programs to improve the quality of care in particular institutions and the implementation of an effective infection control system.

Taking into account the scientific evidence found and guidelines of care safety institutions, it is necessary for each long-term care facility to individually implement interventions to continuously improve the quality of care and patient safety culture at the level of medical and management staff.

It is also necessary to conduct further high-quality research (preferably RCT) analysing the effectiveness of interventions aimed at increasing patient safety in long-term care facilities.

## Figures and Tables

**Table 1:** Inclusion criteria for a systematic review.

Population (P)	Medical staff (including doctors, nurses, physiotherapists), support staff, management staff, patients.
Intervention (I)	Promoting safety culture, interventions aimed at increasing the safety of use of medications and interventions aimed at reducing the level of occupational stress and burnout.
Effects (O)	Patient safety level, safety culture level, care quality level, level of occupational burnout, number of medical errors, number of adverse events.
Type of studies (S)	Systematic reviews, meta-analyses.

**Table 2:** Summary and intervention characteristics of the included studies.

Author/Year	N Studies	Population	Intervention (I) Comparator (C)	Outcomes	Study Findings
**Promoting safety culture in institutions**					
Bukoh 2020 [18] (MA)	9 studies (quasi-experimental, RCT)	Nurses of hospital departments with various specialties, *n* = 1.169 (age from 22 to 38).	(I) Structured patient transfer methods: SBAR, ICCCO, PCH, NHF; (C) No intervention.	Primary: treatment complications, adverse events, medication errors.	The intervention did not reduce post-treatment complications (SMD = −0.15 [95% CI: −0.32; 0.01]) or the incidence of adverse events in patients (SMD = −0.13 [95% CI: −0.26; 0.01]). The intervention reduces medication errors (SMD = −0.07 [95% CI: −0.13; −0.01]).
				Secondary: mistakes in transferring nursing care.	No impact of interventions on the occurrence of errors in transferring nursing care (SMD = −0.47 [95% CI: −1.04; 0.09]).
McCarthy 2018 [19] (SR)	6 studies (pre/post studies without a control group)	Nurses working in acute settings.	(I) Completion of documentation in electronic form and education in the field of its correct completion; (C) NA (no control group).	Improving patient safety and quality of care; completeness of the completed documentation.	Education related to the way of keeping documentation influenced its correctness. Electronic documentation reduces the time needed to complete it (3 months after applying the intervention) from 138.5 h/week to 55.8 h/week (1 study). Positive impact of the quality improvement program in the facility on the change of work management—lower number of medical errors and workplace infections (2 studies).
Snowdon 2016 [24] (MA)	32 studies (prospective and retrospective cohort studies; pre/post studies)	Medical staff (doctors, nurses, paramedics) working in health care facilities.	(I) Clinical supervision by experienced specialists; (C) No intervention or intervention to a lesser extent.	Mortality	The intervention reduces patient mortality (RR = 0.76 [95% CI: 0.60; 0.95]) (14 studies; *n* = 20.474) (low-quality evidence). Subgroup analysis (9 studies; *n* = 6.484) showed a positive effect of the intervention on the reduced risk of death after surgery (RR = 0.68 [95% CI 0.50; 0.93]) (moderate quality evidence).
				Complications after treatment	The intervention reduces the risk of complications after treatment (RR = 0.69 [95% CI: 0.53; 0.89]) (23 studies; *n* = 104.625) (low-quality evidence). Subgroup analysis (3 studies, *n* = 23.609) showed a positive effect of the intervention in reducing the risk of complications after invasive treatment and surgery (RR = 0.33 [95% CI 0.24; 0.46]) (low-quality evidence). No effect of direct intervention during surgery on the reduction of the risk of post-treatment complications (RR = 0.85 [95% CI: 0.67; 1.07]) (16 studies; *n* = 6 6.447) (low-quality evidence).
				Reoperations	No effect of the intervention on the reduced risk of reoperation (RR = 1.16 [95% CI: 0.92; 1.47]) (9 studies; *n* = 10.699). Subgroup analysis (3 studies; *n* = 241) showed a positive effect of the intervention in reducing the risk of switching from less to more severe surgery (RR = 0.39 [95% CI 0.22; 0.69]) (moderate-quality evidence).
Weaver 2013 [26] (SR)	33 studies (27 pre/post studies, 3 time series analyses, 3 RCTs)	Employees of acute settings.	(I) Strategies promoting a safety culture in the facility (training, rounds, comprehensive safety improvement programs). (C) No intervention.	Improving the factors influencing patient safety (e.g., communication in the therapeutic team); change in the level of the safety climate measured with standardized questionnaires (SAQ, HSOPSC, PSCHO).	Training in the field of communication in the therapeutic team has a statistically significant impact on: improving awareness of the importance of maintaining a high level of safety culture in the facility (16 out of 20 studies); reduction of delay in providing care (5 studies); reducing the number of adverse events associated with patient care (7 studies). Rounds improve awareness of the importance of maintaining a high level of safety culture in the facility. Rounds have been shown to change care-related parameters (e.g., improvement in the average number of days since the occurrence of an adverse event) (3 studies). There was no statistically significant effect of lowering the cost of care in the case of rounds (1 study). Comprehensive safety improvement programs contribute to: improving awareness of the importance of maintaining a high level of safety culture in the facility (8 studies); improvement in the care process (2 studies); reduction in the occurrence of medical errors (1 study; 0.56 vs. 0.15, *p* < 0.01).
**Reducing the level of occupational stress, burnout and improving the well-being of medical staff**					
Burton 2017 [20] (MA)	9 studies (1 pre/post control, 6 uncontrolled pre/post, 2 RCT)	Medical staff (nurses, primary care workers, mental disorder specialists).	(I) MBIs: traditional, modified or telephone MBSR, mindfulness-based cognitive attitude training. (C) No intervention.	Level of mindfulness, well-being, anxiety, stress.	MBIs reduces stress among medical staff (7 studies, *n* = 188): combined effect size (r = 0.342 [95% CI 0.202; 0.468]) (medium effect size); combined probability (*p* < 0.0002). The results of individual studies show a moderate effect of the actions depending on the duration of the intervention. Traditional MBSR (duration 4–8 weeks) reduces the level of stress, anxiety and depression. Traditional MBSR improves the sense of occupational burnout and the well-being of medical staff.
Busireddy 2016 [22] (MA)	19 studies (13 cohort studies, 6 RCTs)	Resident doctors working in the following departments: internal medicine, orthopaedics, paediatric, gynaecology, surgery, oncology (*n* = 2030).	(I) Working time limit. (C) No intervention.	The level of occupational burnout (Maslach burnout questionnaire—MBI).	The reduction in the number of hours (up to 80 weeks) worked by residents was associated with a reduction in emotional exhaustion (OR = 0.59 [95% CI 0.45; 0.79]) and a reduction in the level of occupational burnout (MD = −2.70 [95% CI −3.98; −1.41]). Working hours limit did not reduce the number of people with high levels of depersonalization (OR = 0.86 [95% CI 0.64; 1.14]). There was a slight decrease in the mean value of the depersonalization index (MD = −1.43 [95% CI −2.54; −0.31]). No effect of the intervention on the sense of self-realization (MD = 0.99 [95% CI −0.04; −2.02]) and on the number of people with a high rate of self-realization (OR = 1.11 [95% CI 0.74; 1.65]). The intervention reduces the chance of high occupational burnout scores among residents (OR = 0.60 [95% CI 0.37; 0.98]) (2 studies). A positive impact of Balint training on the level of occupational burnout was obtained (2 studies, *n* = 17)—no possibility to determine the size of the effect.
Hill 2016 [23](SR)	9 studies (2 RCTs, 2 non-RCTs, 4 pre/post studies, 1 process evaluation study).	Medical staff working in palliative care facilities.	(I) Relaxation, psychoeducation, support groups, cognitive training, musicotherapy, art therapy. (C) No intervention.	Level of stress, occupational burnout, well-being.	Most of the interventions used had no effect on the endpoints. Additionally, none of the studies showed a greater than low effect on the analyzed endpoints (except for art therapy and musicotherapy, for which a moderate effect was demonstrated).
**Increasing the safety of medication use**					
Kruse 2021 [17] (SR)	14 studies (RCT, pre/post, observational)	Medical staff employed in long-term care facilities.	(I) Health information technology (electronic databases, computerized clinical decision support systems). (C) No intervention/paper documentation.	The amount of time needed for administrative activities, improving the quality of medical records, the level of risk of errors, the level of stress.	Australia—electronic databases: interventions have no effect on reducing the time needed for nurses to complete medical records. There has been an improvement in prescribing adequacy and an increase in the completeness of electronic documentation. Sweden—electronic medication management systems: it has been shown to reduce the stress level among medical staff related to the risk of adverse events and to improve the attitude to conducting administrative activities. Other research—information technology: positive effect on the quality of information provided during staff shifts.
Alldred 2016 [21] (SR)	12 studies (RCT)	Older people (> 65 years old), staying in nursing homes (*n* = 10.953) and medical staff.	(I) Education of prescribers; verification of medication administration (carried out individually for the patient by a nurse, pharmacist or doctor); interdisciplinary discussion meetings; technologies that facilitate clinical decision making. (C) No intervention/care provided by a GP.	Primary: occurrence of adverse medication reactions, hospitalizations, mortality.	Adverse medication reactions: coordinating medication administration by a pharmacist (RR = 1.05 [95% CI 0.66; 1.68]) and the decision support system (aRR = 1.06 [95% CI 0.92; 1.23]) did not affect the occurrence of adverse medication reactions (2 studies). Hospitalization: coordinating medication administration by a pharmacist, combined with informing medical staff about the medications taken by patients, reduces the number of hospitalizations (RR = 0.38 [95% CI 0.15; 0.99]) for those who were alive at the time of follow-up. An intervention analysis for patients who died during the study period showed no effect on this endpoint (RR = 0.58 [95% CI 0.28, 1.21]) (1 study). Nursing education resulted in a reduction in the number of days of hospitalization (1.4 days/person/year [95% CI 1.2; 1.6] (I) vs. 2.3 days/person/year [95% CI 2.1; 2.7]) (aRR = 0.60 [95% CI 0.49; 0.75]) (1 study). Verification of medication use by a pharmacist did not affect the number of hospitalizations (RR = 0.75 [95% CI 0.52; 1.07]) (1 study). Nursing education and interdisciplinary discussion meetings did not impact hospital admissions (RR = 1.02 [95% CI 0.83; 1.26]) (1 study). Mortality: verification of medication use by a pharmacist reduces the number of deaths (4 (I) vs. 14 (C), *p* = 0.028). In the longer term, the number of deaths in both groups became even (26 (I) vs. 28 (C), *p* = ND) (1 study). In another study, verification of medication use by a pharmacist did not reduce the patient’s risk of death (RR = 1.06 [95% CI 0.70; 1.64]) (1 study). Nursing education and interdisciplinary discussion meetings were also not affected by the endpoint (RR = 1.11 [95% CI 0.76; 1.61]) (1 study).
				Secondary: quality of life, problems with taking medications, grounds for the use of medications, costs.	Quality of life: after the intervention consisting in verifying the use of medications by a pharmacist, no difference was found in the quality of life of residents (tested with the SF-12 tool) in the sphere of physical (*p* = 0.09) and mental health (*p* = 0.70) (1 test). Nursing education contributed to a slower deterioration of the quality of life of patients in the study group compared to the control group (−0.038 [95% CI −0.038; −0.022] (I) vs. (−0.072 [95% CI −0.089; −0.055] (C)) (1 study). Grounds for the use of medications (5 studies): the benefit of using the intervention in relation to the control groups was indicated. The intervention consisting of coordinating the administration of medications by a pharmacist combined with informing medical staff about the medications taken by the patient improved the adequacy of medication administration in the study group (mean change in MAI 4.1 [95% CI 2.1; 6.1] (I) vs. MAI 0.4 [95% CI −0.4; 1.2]) (C)) (1 study). The intervention involving verification of the use of medications by a pharmacist reduces the risk of medication misadministration events (37.4% (I) vs. 56% (C), *p* < 0.01) (1 study). Costs (5 studies): the intervention reduces costs in terms of the amount spent on medications (GBP 141.24/person (C) vs. GBP 131.54/person (I)) (3 studies). The intervention reduces costs by GBP 27.47 per capita (1 study).
Marasinghe 2015 [25] (SR)	7 studies (5 RCTs, 2 cohort studies).	People in long-term care facilities (*n* = 13.790) and medical staff.	(I) Computerized clinical decision support systems. (C) No intervention.	Number of incorrect medication orders, number of adverse medication reactions and their severity, number of warnings detected.	No effect of the intervention on the number of adverse events and the adequacy of medication prescription (2 studies). Thanks to the intervention, out of 47,977 registered medication orders, 9414 situations that could adversely affect the adequacy of the medication administration were detected (study). Prescribers were more likely to make optimal treatment decisions (RR = 1.11 [95% CI 1.00; 1.22]) (1 study). The intervention reduced the risk of various types of health damage by 1.7/1000 patients [95% CI 0.2/1000; 3.2/1000; *p* = 0.02]. The intervention improved the quality of care for patients with renal failure (1 study).

aRR—adjusted odds ratio; CI—confidence interval; C—comparator; HSOPSC—Hospital Survey on Patient Safety Culture; I—intervention; ICCCO—identification of the patient and clinical risks, clinical history/presentation, clinical status, care plan and outcomes/goals of care; MA—meta-analysis; MAI—Medication Appropriateness Index; MBI—Maslach Burnout Inventory; MBSi—mindfulness-based interventions; MBSR—Mindfulness Based Stress Reduction; NA—not applicable; ND—no data; NHF—standardized nursing handoff form; PCH—patient-centred handovers; PSCHO—Patient Safety Climate in Healthcare Organizations survey; RCT—randomized controlled trial; SAQ—Safety Attitudes Questionnaire; SBAR—situation, background, assessment, recommendation; SMD—standardized mean difference, SR—systematic review; STOPP-START—Screening Tool of Older Persons’ potentially inappropriate Prescriptions—Screening Tool to Alert doctors to Right Treatment.

## Data Availability

Data available upon reasonable request to the corresponding author.

## Supplementary Materials

The following are available online at https://www.mdpi.com/article/10.3390/ijerph192215354/s1, Table S1: Search strategy Cochrane, Table S2: Search strategy Medline (via PubMed), Table S3: Search strategy Embase (via Ovid), Table S4: List of studies included and excluded after full-text analysis, Table S5: AMSTAR 2 rating.
