# Supplementary material for: Interventions to Increase Patient Safety in Long-Term Care Facilities—Umbrella Review
Source: Int J Environ Res Public Health. 2022 Nov 21;19(22):15354. doi: 10.3390/ijerph192215354 (PMC9691014; doi:10.3390/ijerph192215354)
Supplement: Supplementary file 1 [file ijerph-19-15354-s001.zip › ijerph-2019177-supplementary.pdf]

Table S1. Search strategy Cochrane

ID	Keyword	Result	Update result
#1	MeSH descriptor: [Health Personnel] explode all trees	9059	10297
#2	MeSH descriptor: [Medical Staff] explode all trees	331	344
#3	MeSH descriptor: [Nursing Staff] explode all trees	650	683
#4	MeSH descriptor: [Physicians] explode all trees	2078	2379
#5	MeSH descriptor: [General Practitioners] explode all trees	280	332
#6	MeSH descriptor: [Physical Therapists] explode all trees	119	153
#7	MeSH descriptor: [Nurse Administrators] explode all trees	28	29
#8	("health personnel"):ti,ab,kw	3644	3940
#9	("medical staff"):ti,ab,kw	1471	1610
#10	(nurse*):ti,ab,kw	26663	29011
#11	("nursing staff"):ti,ab,kw	2293	2509
#12	(physician*):ti,ab,kw	45163	48715
#13	(doctor*):ti,ab,kw	15110	16422
#14	("general practitioner*"):ti,ab,kw	7222	7652
#15	(physiotherapist*):ti,ab,kw	5318	6057
#16	("physical therapists"):ti,ab,kw	1814	2038
#17	(manager*):ti,ab,kw	3479	3823
#18	(chief):ti,ab,kw	1185	1343
#19	#1 OR #2 OR #3 OR #4 OR #5 OR #6 OR #7 OR #8 OR #9 OR #10 OR #11 OR #12 OR #13 OR #14 OR #15 OR #16 OR #17 OR #18	96670	105427
#20	MeSH descriptor: [Patient Safety] explode all trees	659	718
#21	MeSH descriptor: [Safety Management] explode all trees	173	176
#22	MeSH descriptor: [Patient Compliance] explode all trees	12131	12652
#23	MeSH descriptor: [Continuity of Patient Care] explode all trees	25532	28607
#24	MeSH descriptor: [Physician-Patient Relations] explode all trees	1403	1465
#25	MeSH descriptor: [Patient-Centered Care] explode all trees	747	837
#26	MeSH descriptor: [Medication Adherence] explode all trees	2440	2716
#27	MeSH descriptor: [Patient Satisfaction] explode all trees	12271	12775
#28	MeSH descriptor: [Quality Improvement] explode all trees	739	817
#29	("patient safety"):ti,ab,kw	7356	7935
#30	("safety management"):ti,ab,kw	239	247
#31	("safety culture"):ti,ab,kw	81	89
#32	("quality of health care"):ti,ab,kw	1019	1063
#33	("patient compliance"):ti,ab,kw	20654	21476
#34	("continuity of patient care"):ti,ab,kw	678	697

#35	("physician-patient relations"):ti,ab,kw	1456	1506
#36	("patient-centered care"):ti,ab,kw	1062	1150
#37	("medication adherence"):ti,ab,kw	5776	6430
#38	("patient satisfaction"):ti,ab,kw	28169	30598
#39	("patient experience"):ti,ab,kw	3389	3928
#40	("quality improvement"):ti,ab,kw	3165	3473
#41	#20 OR #21 OR #22 OR #23 OR #24 OR #25 OR #26 OR #27 OR #28 OR #29 OR #30 OR #31 OR #32 OR #33 OR #34 OR #35 OR #36 OR #37 OR #38 OR #39 OR #40	90000	98105
#42	MeSH descriptor: [Medical Errors] explode all trees	2785	2942
#43	MeSH descriptor: [Malpractice] explode all trees	21	23
#44	("medical error*"):ti,ab,kw	955	1019
#45	("human error*"):ti,ab,kw	69	83
#46	(malpractice):ti,ab,kw	76	83
#47	("iatrogenic disease*"):ti,ab,kw	299	325
#48	("serious incident*"):ti,ab,kw	16	22
#49	(service* OR system* OR communication* OR organisation* OR organization*):ti,ab,kw	303453	331246
#50	(weak* OR fail*):ti,ab,kw	139588	150364
#51	#49 AND #50	32009	34922
#52	(adverse OR avoidable OR preventable OR unsafe OR safet*):ti,ab,kw	608783	654740
#53	(event* OR outcome* OR complication* OR death OR effect* OR reaction* OR accident* OR injur*):ti,ab,kw	1289537	1389997
#54	#52 AND #53	531861	572702
#55	(medical* OR diagnostic OR therapeautic OR administration OR dispensing OR prescri*):ti,ab,kw	538878	572593
#56	(error* OR mistake* OR fault):ti,ab,kw	21115	23428
#57	#55 AND #56	7809	8610
#58	(patient):ti,ab,kw	989539	1064502
#59	(risk OR incident OR accident):ti,ab,kw	338211	369129
#60	#58 AND #59	237254	259089
#61	#42 OR #43 OR #44 OR #45 OR #46 OR #47 OR #48 OR #51 OR #54 OR #57 OR #60	653412	705210
#62	#19 AND #41 AND #61	7646	7646
#63	19 AND #41 AND #61 with Cochrane Library publication date Between Jan 2010 and Mar 2021, in Cochrane Reviews<br><br>Filters applied in search update: publication date Between 14/03/2021 and 24/05/2022, in Cochrane Reviews	179	20

Table S2. Search strategy Medline (via PubMed)

ID	Keyword	Result	Update result
#1	Search: Health Personnel[MeSH Terms]	532953	582430
#2	Search: Medical Staff[MeSH Terms]	27873	28533
#3	Search: Nursing Staff[MeSH Terms]	67196	69039
#4	Search: Physicians[MeSH Terms]	147916	166003
#5	Search: General Practitioners[MeSH Terms]	8316	9693
#6	Search: Physical Therapists[MeSH Terms]	2080	2702
#7	Search: Nurse Administrators[MeSH Terms]	13394	13876
#8	Search: "health personnel"[Title/Abstract]	8108	8796
#9	Search: "medical staff"[Title/Abstract]	13021	14501
#10	Search: nurse*[Title/Abstract]	286442	305706
#11	Search: "nursing staff"[Title/Abstract]	12747	13810
#12	Search: physician*[Title/Abstract]	408241	435148
#13	Search: doctor*[Title/Abstract]	133209	142938
#14	Search: "general practitioner*"[Title/Abstract]	52042	54984
#15	Search: physiotherapist*[Title/Abstract]	8568	9742
#16	Search: "physical therapists"[Title/Abstract]	4532	5070
#17	Search: manager*[Title/Abstract]	57021	63440
#18	Search: chief[Title/Abstract]	28131	30781
#19	Search: (((((((((((((((((Health Personnel[MeSH Terms]) OR (Medical Staff[MeSH Terms])) OR (Nursing Staff[MeSH Terms])) OR (Physicians[MeSH Terms])) OR (General Practitioners[MeSH Terms])) OR (Physical Therapists[MeSH Terms])) OR (Nurse Administrators[MeSH Terms])) OR ("health personnel"[Title/Abstract])) OR ("medical staff"[Title/Abstract])) OR (nurse*[Title/Abstract])) OR ("nursing staff"[Title/Abstract])) OR (physician*[Title/Abstract])) OR (doctor*[Title/Abstract])) OR ("general practitioner*"[Title/Abstract])) OR (physiotherapist*[Title/Abstract])) OR ("physical therapists"[Title/Abstract])) OR (manager*[Title/Abstract])) OR (chief[Title/Abstract])	1219348	1310922
#20	Search: Patient Safety[MeSH Terms]	22025	24480
#21	Search: Safety Management[MeSH Terms]	20557	21155
#22	Search: Patient Compliance[MeSH Terms]	79911	84128
#23	Search: Continuity of Patient Care[MeSH Terms]	253952	276501
#24	Search: Physician-Patient Relations[MeSH Terms]	73640	75489
#25	Search: Patient-Centered Care[MeSH Terms]	21224	23176
#26	Search: Medication Adherence[MeSH Terms]	21917	24616

#27	Search: Patient Satisfaction[MeSH Terms]	92380	97411
#28	Search: Quality Improvement[MeSH Terms]	27918	31995
#29	Search: "patient safety"[Title/Abstract]	33391	37314
#30	Search: "safety management"[Title/Abstract]	1495	1787
#31	Search: "safety culture"[Title/Abstract]	2275	2621
#32	Search: "quality of health care"[Title/Abstract]	4735	5198
#33	Search: "patient compliance"[Title/Abstract]	10103	10882
#34	Search: "continuity of patient care"[Title/Abstract]	558	661
#35	Search: "physician-patient relations"[Title/Abstract]	1551	1641
#36	Search: "patient-centered care"[Title/Abstract]	5613	6688
#37	Search: "medication adherence"[Title/Abstract]	11847	13527
#38	Search: "patient satisfaction"[Title/Abstract]	38946	43260
#39	Search: "patient experience"[Title/Abstract]	6886	8383
#40	Search: "quality improvement"[Title/Abstract]	41566	47674
#41	Search: ((((((((((((((((((Patient Safety[MeSH Terms]) OR (Safety Management[MeSH Terms])) OR (Patient Compliance[MeSH Terms])) OR (Continuity of Patient Care[MeSH Terms])) OR (Physician-Patient Relations[MeSH Terms])) OR (Patient-Centered Care[MeSH Terms])) OR (Medication Adherence[MeSH Terms])) OR (Patient Satisfaction[MeSH Terms])) OR (Quality Improvement[MeSH Terms])) OR ("patient safety"[Title/Abstract])) OR ("safety management"[Title/Abstract])) OR ("safety culture"[Title/Abstract])) OR ("quality of health care"[Title/Abstract])) OR ("patient compliance"[Title/Abstract])) OR ("continuity of patient care"[Title/Abstract])) OR ("physician-patient relations"[Title/Abstract])) OR ("patient-centered care"[Title/Abstract])) OR ("medication adherence"[Title/Abstract])) OR ("patient satisfaction"[Title/Abstract])) OR ("patient experience"[Title/Abstract])) OR ("quality improvement"[Title/Abstract])	636297	686638
#42	Search: Medical Errors[MeSH Terms]	115395	120058
#43	Search: Malpractice[MeSH Terms]	31798	32213
#44	Search: "medical error*"[Title/Abstract]	5308	5740
#45	Search: "human error*"[Title/Abstract]	2461	2819
#46	Search: malpractice[Title/Abstract]	10359	10697
#47	Search: "iatrogenic disease*"[Title/Abstract]	1343	1378
#48	Search: "serious incident*"[Title/Abstract]	233	250
#49	Search: service*[Title/Abstract] OR system*[Title/Abstract] OR communication*[Title/Abstract] OR organisation*[Title/Abstract] OR organization*[Title/Abstract]	4797762	5239889
#50	Search: weak*[Title/Abstract] OR fail*[Title/Abstract]	1508683	1618105
#51	Search: (service*[Title/Abstract] OR system*[Title/Abstract] OR communication*[Title/Abstract] OR organisation*[Title/Abstract] OR	303926	332312

	organization*[Title/Abstract]) AND (weak*[Title/Abstract] OR fail*[Title/Abstract])		
#52	Search: adverse[Title/Abstract] OR avoidable[Title/Abstract] OR preventable[Title/Abstract] OR unsafe[Title/Abstract] OR safet*[Title/Abstract]	1019678	1146300
#53	Search: event*[Title/Abstract] OR outcome*[Title/Abstract] OR complication*[Title/Abstract] OR death[Title/Abstract] OR effect*[Title/Abstract] OR reaction*[Title/Abstract] OR accident*[Title/Abstract] OR injur*[Title/Abstract]	11228524	12146537
#54	Search: (adverse[Title/Abstract] OR avoidable[Title/Abstract] OR preventable[Title/Abstract] OR unsafe[Title/Abstract] OR safet*[Title/Abstract]) AND (event*[Title/Abstract] OR outcome*[Title/Abstract] OR complication*[Title/Abstract] OR death[Title/Abstract] OR effect*[Title/Abstract] OR reaction*[Title/Abstract] OR accident*[Title/Abstract] OR injur*[Title/Abstract])	811803	914634
#55	Search: medical*[Title/Abstract] OR diagnostic[Title/Abstract] OR therapeautic[Title/Abstract] OR administration[Title/Abstract] OR dispensing[Title/Abstract] OR prescri*[Title/Abstract]	2953321	3191645
#56	Search: error*[Title/Abstract] OR mistake*[Title/Abstract] OR fault[Title/Abstract]	351854	384100
#57	Search: (medical*[Title/Abstract] OR diagnostic[Title/Abstract] OR therapeautic[Title/Abstract] OR administration[Title/Abstract] OR dispensing[Title/Abstract] OR prescri*[Title/Abstract]) AND (error*[Title/Abstract] OR mistake*[Title/Abstract] OR fault[Title/Abstract])	53140	58285
#58	Search: patient[Title/Abstract]	2459635	2666943
#59	Search: risk[Title/Abstract] OR incident[Title/Abstract] OR accident[Title/Abstract]	2328428	2587565
#60	Search: (patient[Title/Abstract]) AND (risk[Title/Abstract] OR incident[Title/Abstract] OR accident[Title/Abstract])	373651	416979
#61	Search: (((((((((Medical Errors[MeSH Terms]) OR (Malpractice[MeSH Terms])) OR ("medical error*"[Title/Abstract])) OR ("human error*"[Title/Abstract])) OR (malpractice[Title/Abstract])) OR ("iatrogenic disease*"[Title/Abstract])) OR ("serious incident*"[Title/Abstract])) OR ((service*[Title/Abstract] OR system*[Title/Abstract] OR communication*[Title/Abstract] OR organisation*[Title/Abstract] OR organization*[Title/Abstract]) AND (weak*[Title/Abstract] OR fail*[Title/Abstract]))) OR ((adverse[Title/Abstract] OR avoidable[Title/Abstract] OR preventable[Title/Abstract] OR unsafe[Title/Abstract] OR safet*[Title/Abstract]) AND (event*[Title/Abstract] OR outcome*[Title/Abstract] OR complication*[Title/Abstract] OR death[Title/Abstract] OR effect*[Title/Abstract] OR reaction*[Title/Abstract] OR accident*[Title/Abstract] OR injur*[Title/Abstract]))) OR ((medical*[Title/Abstract] OR diagnostic[Title/Abstract] OR therapeautic[Title/Abstract] OR administration[Title/Abstract] OR	1576742	1747939

	dispensing[Title/Abstract] OR prescri*[Title/Abstract]) AND (error*[Title/Abstract] OR mistake*[Title/Abstract] OR fault[Title/Abstract]))) OR ((patient[Title/Abstract]) AND (risk[Title/Abstract] OR incident[Title/Abstract] OR accident[Title/Abstract]))		
#62	Search: ((((((((((((((((((Health Personnel[MeSH Terms]) OR (Medical Staff[MeSH Terms])) OR (Nursing Staff[MeSH Terms])) OR (Physicians[MeSH Terms])) OR (General Practitioners[MeSH Terms])) OR (Physical Therapists[MeSH Terms])) OR (Nurse Administrators[MeSH Terms])) OR ("health personnel"[Title/Abstract])) OR ("medical staff"[Title/Abstract])) OR (nurse*[Title/Abstract])) OR ("nursing staff"[Title/Abstract])) OR (physician*[Title/Abstract])) OR (doctor*[Title/Abstract])) OR ("general practitioner*"[Title/Abstract])) OR (physiotherapist*[Title/Abstract])) OR ("physical therapists"[Title/Abstract])) OR (manager*[Title/Abstract])) OR (chief[Title/Abstract])) AND ((((((((((((((((((((Patient Safety[MeSH Terms]) OR (Safety Management[MeSH Terms])) OR (Patient Compliance[MeSH Terms])) OR (Continuity of Patient Care[MeSH Terms])) OR (Physician-Patient Relations[MeSH Terms])) OR (Patient-Centered Care[MeSH Terms])) OR (Medication Adherence[MeSH Terms])) OR (Patient Satisfaction[MeSH Terms])) OR (Quality Improvement[MeSH Terms])) OR ("patient safety"[Title/Abstract])) OR ("safety management"[Title/Abstract])) OR ("safety culture"[Title/Abstract])) OR ("quality of health care"[Title/Abstract])) OR ("patient compliance"[Title/Abstract])) OR ("continuity of patient care"[Title/Abstract])) OR ("physician-patient relations"[Title/Abstract])) OR ("patient-centered care"[Title/Abstract])) OR ("medication adherence"[Title/Abstract])) OR ("patient satisfaction"[Title/Abstract])) OR ("patient experience"[Title/Abstract])) OR ("quality improvement"[Title/Abstract]))) AND ((((((((((Medical Errors[MeSH Terms]) OR (Malpractice[MeSH Terms])) OR ("medical error*"[Title/Abstract])) OR ("human error*"[Title/Abstract])) OR (malpractice[Title/Abstract])) OR ("iatrogenic disease*"[Title/Abstract])) OR ("serious incident*"[Title/Abstract])) OR ((service*[Title/Abstract] OR system*[Title/Abstract] OR communication*[Title/Abstract] OR organisation*[Title/Abstract] OR organization*[Title/Abstract]) AND (weak*[Title/Abstract] OR fail*[Title/Abstract]))) OR ((adverse[Title/Abstract] OR avoidable[Title/Abstract] OR preventable[Title/Abstract] OR unsafe[Title/Abstract] OR safet*[Title/Abstract]) AND (event*[Title/Abstract] OR outcome*[Title/Abstract] OR complication*[Title/Abstract] OR death[Title/Abstract] OR effect*[Title/Abstract] OR reaction*[Title/Abstract] OR accident*[Title/Abstract] OR injur*[Title/Abstract]))) OR ((medical*[Title/Abstract] OR diagnostic[Title/Abstract] OR therapeautic[Title/Abstract] OR administration[Title/Abstract] OR dispensing[Title/Abstract] OR prescri*[Title/Abstract]) AND (error*[Title/Abstract] OR mistake*[Title/Abstract] OR fault[Title/Abstract]))) OR ((patient[Title/Abstract]) AND	30203	33465

	(risk[Title/Abstract] OR incident[Title/Abstract] OR accident[Title/Abstract])))		
#63	Search: (((((((((((((((((Health Personnel[MeSH Terms]) OR (Medical Staff[MeSH Terms])) OR (Nursing Staff[MeSH Terms])) OR (Physicians[MeSH Terms])) OR (General Practitioners[MeSH Terms])) OR (Physical Therapists[MeSH Terms])) OR (Nurse Administrators[MeSH Terms])) OR ("health personnel"[Title/Abstract])) OR ("medical staff"[Title/Abstract])) OR (nurse*[Title/Abstract])) OR ("nursing staff"[Title/Abstract])) OR (physician*[Title/Abstract])) OR (doctor*[Title/Abstract])) OR ("general practitioner*"[Title/Abstract])) OR (physiotherapist*[Title/Abstract])) OR ("physical therapists"[Title/Abstract])) OR (manager*[Title/Abstract])) OR (chief[Title/Abstract])) AND (((((((((((((((((((Patient Safety[MeSH Terms]) OR (Safety Management[MeSH Terms])) OR (Patient Compliance[MeSH Terms])) OR (Continuity of Patient Care[MeSH Terms])) OR (Physician-Patient Relations[MeSH Terms])) OR (Patient-Centered Care[MeSH Terms])) OR (Medication Adherence[MeSH Terms])) OR (Patient Satisfaction[MeSH Terms])) OR (Quality Improvement[MeSH Terms])) OR ("patient safety"[Title/Abstract])) OR ("safety management"[Title/Abstract])) OR ("safety culture"[Title/Abstract])) OR ("quality of health care"[Title/Abstract])) OR ("patient compliance"[Title/Abstract])) OR ("continuity of patient care"[Title/Abstract])) OR ("physician-patient relations"[Title/Abstract])) OR ("patient-centered care"[Title/Abstract])) OR ("medication adherence"[Title/Abstract])) OR ("patient satisfaction"[Title/Abstract])) OR ("patient experience"[Title/Abstract])) OR ("quality improvement"[Title/Abstract]))) AND ((((((((((Medical Errors[MeSH Terms]) OR (Malpractice[MeSH Terms])) OR ("medical error*"[Title/Abstract])) OR ("human error*"[Title/Abstract])) OR (malpractice[Title/Abstract])) OR ("iatrogenic disease*"[Title/Abstract])) OR ("serious incident*"[Title/Abstract])) OR ((service*[Title/Abstract] OR system*[Title/Abstract] OR communication*[Title/Abstract] OR organisation*[Title/Abstract] OR organization*[Title/Abstract]) AND (weak*[Title/Abstract] OR fail*[Title/Abstract]))) OR ((adverse[Title/Abstract] OR avoidable[Title/Abstract] OR preventable[Title/Abstract] OR unsafe[Title/Abstract] OR safet*[Title/Abstract]) AND (event*[Title/Abstract] OR outcome*[Title/Abstract] OR complication*[Title/Abstract] OR death[Title/Abstract] OR effect*[Title/Abstract] OR reaction*[Title/Abstract] OR accident*[Title/Abstract] OR injur*[Title/Abstract]))) OR ((medical*[Title/Abstract] OR diagnostic[Title/Abstract] OR therapeautic[Title/Abstract] OR administration[Title/Abstract] OR	819	155

	dispensing[Title/Abstract] OR prescri*[Title/Abstract]) AND (error*[Title/Abstract] OR mistake*[Title/Abstract] OR fault[Title/Abstract]))) OR ((patient[Title/Abstract]) AND (risk[Title/Abstract] OR incident[Title/Abstract] OR accident[Title/Abstract]))) Filters: Meta-Analysis, Systematic Review, English, from 2010 – 2021<br><br>Filters applied in search update: Meta-Analysis, Systematic Review, English, from 2021/3/14 - 2022/5/24		

Table S3. Search strategy Embase (via Ovid)

ID	Keyword	Result	Update result
#1	exp medical staff/	33935	35864
#2	exp nursing staff/	63905	66263
#3	exp physician/	778278	846601
#4	exp general practitioner/	98790	105148
#5	exp physiotherapist/	21746	24116
#6	exp nurse administrator/	13649	14178
#7	health personnel.ab,kw,ti.	6107	5791
#8	medical staff.ab,kw,ti.	17431	19096
#9	nurse*.ab,kw,ti.	313358	335135
#10	nursing staff.ab,kw,ti.	18388	19862
#11	physician*.ab,kw,ti.	536226	573162
#12	doctor*.ab,kw,ti.	174258	186937
#13	general practitioner.ab,kw,ti.	23559	24817
#14	physiotherapist*.ab,kw,ti.	17006	18740
#15	physical therapists.ab,kw,ti.	6105	6761
#16	manager*.ab,kw,ti.	66390	72436
#17	chief.ab,kw,ti.	34691	38618
#18	1 or 2 or 3 or 4 or 5 or 6 or 7 or 8 or 9 or 10 or 11 or 12 or 13 or 14 or 15 or 16 or 17	1581372	1703199
#19	exp patient safety/	132831	147933
#20	exp patient compliance/	160329	172132
#21	exp doctor patient relationship/	4601	6868
#22	exp medication compliance/	34850	40216
#23	exp patient satisfaction/	144389	155616
#24	exp total quality management/	70756	79006
#25	patient safety.ab,kw,ti.	49033	53605
#26	safety management.ab,kw,ti.	2557	2830
#27	safety culture.ab,kw,ti.	3078	3452
#28	quality of health care.ab,kw,ti.	10203	10854
#29	patient compliance.ab,kw,ti.	15875	17058
#30	continuity of patient care.ab,kw,ti.	880	974
#31	physician-patient relations.ab,kw,ti.	1235	1300
#32	patient-centered care.ab,kw,ti.	7481	8597
#33	medication adherence.ab,kw,ti.	19595	21855
#34	patient satisfaction.ab,kw,ti.	57255	62719
#35	patient experience.ab,kw,ti.	12277	14476

#36	quality improvement.ab,kw,ti.	64956	73022
#37	19 or 20 or 21 or 22 or 23 or 24 or 25 or 26 or 27 or 28 or 29 or 30 or 31 or 32 or 33 or 34 or 35 or 36	572450	628312
#38	exp medical error/	140876	152287
#39	exp malpractice/	25820	26270
#40	medical error*.ab,kw,ti.	7385	7876
#41	human error*.ab,kw,ti.	3793	4205
#42	malpractice.ab,kw,ti.	9082	9278
#43	iatrogenic disease*.ab,kw,ti.	919	950
#44	serious incident*.ab,kw,ti.	413	452
#45	(service* or system* or communication* or organisation* or organization*).ab,kw,ti.	5538833	5939973
#46	(weak* or fail*).ab,kw,ti.	1906795	2020107
#47	45 and 46	408061	434193
#48	(adverse or avoidable or preventable or unsafe or safet*).ab,kw,ti.	1517759	1662450
#49	(event* or outcome* or complication* or death or effect* or reaction* or accident* or injur*).ab,kw,ti.	12991137	13955264
#50	48 and 49	1244799	1366230
#51	(medical* or diagnostic or therapeautic or administration or dispensing or prescri*).ab,kw,ti.	3797447	4086438
#52	(error* or mistake* or fault).ab,kw,ti.	422871	460622
#53	51 and 52	79718	84385
#54	patient.ab,kw,ti.	3402763	3668160
#55	(risk or incident or accident).ab,kw,ti.	3306607	3606746
#56	54 and 55	633106	694992
#57	38 or 39 or 40 or 41 or 42 or 43 or 44 or 47 or 50 or 53 or 56	2328996	2538609
#58	18 and 37 and 57	41049	44274
#59	limit 58 to ((meta analysis or "systematic review") and (english) and yr="2010-Current")<br><br>Filters applied in search update: ((meta analysis or "systematic review") and (english) and yr="2021-Current")	1225	270

Table S4. List of studies included and excluded after full-text analysis

Lp.	Authors, Title, Journal	Full text status	Reason for exclusion
1	Kruse, C.S.; Mileski, M.; Syal, R.; MacNeil, L.; Chabarria, E.; Basch, C. Evaluating the relationship between health information technology and safer-prescribing in the long-term care setting: A systematic review. *Technol Health Care*. **2021**, *29(1)*, 1-14.	**Included**	–
2	Bukoh, M.X.; Siah C.R. A systematic review on the structured handover interventions between nurses in improving patient safety outcomes. *J Nurs Manag*. **2020**, *28(3)*, 744-755.	**Included**	–
3	McCarthy, B.; Fitzgerald, S.; O'Shea, M.; Condon, C.; Hartnett-Collins, G.; Clancy, M.; Sheehy, A.; Denieffe, S.; Bergin, M.; Savage E. Electronic nursing documentation interventions to promote or improve patient safety and quality care: A systematic review. *J Nurs Manag*. **2019**, *27(3)*, 491-501.	**Included**	–
4	Burton, A.; Burgess, C.; Dean, S.; Koutsopoulou, G.Z.; Hugh-Jones, S. How Effective are Mindfulness-Based Interventions for Reducing Stress Among Healthcare Professionals? A Systematic Review and Meta-Analysis. *Stress Health*. **2017**, *33(1)*: 3-13.	**Included**	–
5	Alldred, D.P.; Kennedy, M.C.; Hughes, C.; Chen, T.F.; Miller P. et al. Interventions to optimise prescribing for older people in care homes. *Cochrane Database Syst Rev*. **2016**, *2(2)*, CD009095.	**Included**	–
6	Busireddy, K.R.; Miller, J.A.; Ellison, K.; Ren, V.; Qayyum, R.; Panda M. Efficacy of interventions to reduce resident physician burnout: A systematic review. *J Grad Med Educ*. **2017**, *9(3)*, 294-301.	**Included**	–
7	Hill, R.C.; Dempster, M.; Donnelly, M.; McCorry N.K. Improving the wellbeing of staff who work in palliative care settings: A systematic review of psychosocial interventions. *Palliat Med*. **2016**, *30(9)*, 825-833.	**Included**	–
8	Snowdon, D.A.; Hau, R.; Leggat, S.G.; Taylor N.F. Does clinical supervision of health professionals improve patient safety? A systematic review and meta-analysis. *Int J Qual Health Care*. **2016**, *28(4)*, 447-455.	**Included**	–
9	Marasinghe, K.M. Computerised clinical decision support systems to improve medication safety in long-term care homes: a systematic review. *BMJ Open*. **2015**, *5(5)*, e006539.	**Included**	–
10	Weaver, S.J.; Lubomski, L.H.; Wilson, R.F.; Pfoh, E.R.; Martinez, K.A.; Dy, S.M. Promoting a culture of safety as a patient safety strategy: a systematic review. *Ann Intern Med*. **2013**, *158*, 369-374.	**Included**	–
11	Aaron, M.; Webb, A.; Luhanga U. A Narrative Review of Strategies to Increase Patient Safety Event Reporting by Residents. *J Grad Med Educ*. **2020**, *12(4)*, 415-424.	Excluded	S
12	Berglas, N.F.; Battistelli, M.F; Nicholson W.K.; Sobota, M.; Urman, R.D.; Roberts S.C.M. The effect of facility characteristics on patient safety, patient experience, and service availability for procedures in non-hospital-affiliated outpatient settings: A systematic review. *PLoS One*. **2018**, *13(1)*, e0190975.	Excluded	P, I

13	Bilotta, F.F.; Werner, S.M.; Bergese, S.D.; Rosa, G. Impact and implementation of simulation-based training for safety. *The Scientific World Journal*. **2013**, 652956.	Excluded	P, I, S
14	Boyd, J.; Wu, G.; Stelfox, H. The impact of checklists on inpatient safety outcomes: A systematic review of randomized controlled trials. *J Hosp Med*. **2017**, *12(8)*, 675-682.	Excluded	P
15	Campbell, A.R.; Layne, D.; Scott, E.; Wei H. Interventions to promote teamwork, delegation and communication among registered nurses and nursing assistants: An integrative review. *J Nurs Manag*. **2020**, *28(7)*, 1465-1472.	Excluded	P
16	Fischer, S.A.; Jones, J.; Verran, J.A. Consensus achievement of leadership, organisational and individual factors that influence safety climate: Implications for nursing management. *J Nurs Manag*. **2018**, *26(1)*, 50-58.	Excluded	I
17	Havyer, R.D.A.; Wingo, M.T.; Comfere, N.I.; Nelson, D.R.; Halvorsen, A.J.; McDonald, F.S.; Reed D.A. Teamwork assessment in internal medicine: A systematic review of validity evidence and outcomes. *J Gen Intern Med*. **2014**, *29(6)*, 894-910.	Excluded	I
18	Keen, J.; Abdulwahid, M.A.; King, N.; Wright, J.M.; Randell, R.; Gardner, P.; Waring, J.; Longo, R.; Nikolova, S.; Sloan C.; et al. Effects of interorganisational information technology networks on patient safety: A realist synthesis. *BMJ Open*. **2020**, *10(10)*, e036608.	Excluded	I
19	Li, R.; Zaidi, S.T.R.; Chen, T.; Castelino, R. Effectiveness of interventions to improve adverse drug reaction reporting by healthcare professionals over the last decade: A systematic review. *Pharmacoepidemiol Drug Saf*. **2020**, *29(1)*, 1-8.	Excluded	P, S
20	McCulloch, P.; Rathbone, J.; Catchpole, K. Interventions to improve teamwork and communications among healthcare staff. *Br J Surg*. **2011**, *98(4)*, 469-79.	Excluded	P, I
21	McDowell, D. S.; McComb, S. A. Safety checklist briefings: a systematic review of the literature. *AORN J*. **2014**, *99(1)*, 125-137.e13.	Excluded	P, S
22	Ock, M.; Lim, S.Y.; Jo, M.W.; Lee S.I. Frequency, Expected Effects, Obstacles, and Facilitators of Disclosure of Patient Safety Incidents: A Systematic Review. *J Prev Med Public Health*. **2017**, *50(2)*, 68-82..	Excluded	P, I, S
23	Richardson, A.; Storr, J. Patient safety: a literature [corrected] review on the impact of nursing empowerment, leadership and collaboration. *Int Nurs Rev*. **2010**, *57(1)*, 12-21.	Excluded	S
24	Rouleau, G.; Gagnon, M.P.; Côté, J.; Payne-Gagnon, J.; Hudson, E.; Dubois, C.A. Impacts of information and communication technologies on nursing care: Results of an overview of systematic reviews. *J Med Internet Res*. **2017**, *19(4)*, e122.	Excluded	I
25	Sarazine, J.; Heitschmidt, M.; Vondracek, H.; Sarris, S.; Marcinkowski, N.; Kleinpell, R. Mindfulness Workshops Effects on Nurses' Burnout, Stress, and Mindfulness Skills. *Holist Nurs Pract*. **2021**, *35(1)*, 10-18.	Excluded	S

26	Schmutz, J.; Manser, T. Do team processes really have an effect on clinical performance? A systematic literature review. *Br J Anaesth*. **2013**, *110(4)*, 529-544.	Excluded	P
27	Squires, A.; Murali, K.P.; Greenberg S.A.; Herrmann, L.L.; D'amico, C.O. A Scoping Review of the Evidence About the Nurses Improving Care for Healthsystem Elders (NICHE) Program. *Gerontologist*. **2021**, *61(3)*, e75-e84.	Excluded	I, S
28	Stavropoulou, C.; Doherty, C.; Tosey, P. How Effective Are Incident-Reporting Systems for Improving Patient Safety? A Systematic Literature Review. *Milbank Q*. **2015**, *93(4)*, 826-866.	Excluded	S
29	Vaismoradi, M.; Tella, S.; Logan, A.; Khakurel, J.; Vizcaya-Moreno F. Nurses' Adherence to Patient Safety Principles: A Systematic Review. *Int J Environ Res Public Health*. **2020**, *17(6)*, 2028.	Excluded	I
30	Zohar, D.; Werber, Y.; Marom, R.; Curlau, B.; Blondheim O. Modifying head nurse messages during daily conversations as leverage for safety climate improvement: A randomised field experiment. *BMJ Qual Saf*. **2017**, *26(8)*, 653-662.	Excluded	S
31	Amer, F.; Hammoud, S.; Khatatbeh, H.; Lohner, S.; Boncz, I.; Endrei, D. The deployment of balanced scorecard in health care organizations: is it beneficial? A systematic review. *BMC Health Serv Res*. **2022**, *22(1)*, 65.	Excluded	I, S
32	Berg, M.N.; Ngune, I.; Schofield, P.; Grech, L.; Juraskova I.; Strasser, M.; Butt, Z.; Halkett, G.K.B. Effectiveness of online communication skills training for cancer and palliative care health professionals: A systematic review. *Psychooncology*. **2021**, *30(9)*, 1405-1419.	Excluded	P, S
33	Lee, M.; Lee, N.J.; Seo, H.J.; Jang, H.; Kim, S.M. Interventions to Engage Patients and Families in Patient Safety: A Systematic Review. *West J Nurs Res*. **2021**, *43(10)*, 972-983.	Excluded	P, S
34	Lee, S.E.; Morse, B.L.; Kim, N.W. Patient safety educational interventions: A systematic review with recommendations for nurse educators. *Nurs Open*. **2022**, *9(4)*, 1967-1979.	Excluded	P
35	O'Brien, N.; Shaw, A.; Flott, K.; Leatherman, S.; Durkin, M. Safety in fragile, conflict-affected, and vulnerable settings: An evidence scanning approach for identifying patient safety interventions. *J Glob Health*. **2022**, *12*, 04018.	Excluded	S
36	Serou, N.; Sahota, L.M.; Husband, A.K.; Forrest, S.P.; Slight, R.D.; Slight; S.P. Learning from safety incidents in high-reliability organizations: a systematic review of learning tools that could be adapted and used in healthcare. *Int J Qual Health Care*. **2021**, *33(1)*, mzab046	Excluded	S
37	Woo, M.W.J.; Avery, M.J. Nurses' experiences in voluntary error reporting: An integrative literature review. *Int J Nurs Sci*. **2021**, *8(4)*, 453-469.	Excluded	I

Reason for exclusion: I – inadequate intervention; P – inadequate population; S – inadequate methodology.

AMSTAR2

The systematic reviews included in the analysis received the following ratings:

- high – Snowdon 2016, Alldred 2016, Weaver 2013;
- low – Kruse 2021, Bukoh 2020, McCarthy 2018, Burton 2017;
- critically low – Busireddy 2016, Hill 2016, Marasinghe 2015.

Table S5. AMSTAR 2 rating

Publication	Item 2	Item 4	Item 7	Item 9	Item 11	Item 13	Item 15	Overall rating
Kruse 2021 (SR)	Yes	Partial Yes	Partial Yes	Partial Yes	-	No	-	**Low**
Bukoh 2020 (MA)	Partial Yes	Yes	No	Yes	Yes	Yes	Yes	**Low**
McCarthy 2018 (SR)	Partial Yes	Yes	No	Yes	-	Yes	-	**Low**
Burton 2017 (MA)	Partial Yes	Yes	No	Partial Yes	Yes	Yes	Yes	**Low**
Busireddy 2016 (MA)	No	Yes	No	No	Yes	No	No	**Critically Low**
Hill 2016 (SR)	Partial Yes	Partial Yes	Partial Yes	No	-	No	-	**Critically Low**
Snowdon 2016 (MA)	Yes	Yes	Yes	Yes	Yes	Yes	Yes	**High**
Alldred 2016 (SR)	Yes	Yes	Yes	Yes	-	Yes	-	**High**
Marasinghe 2015 (SR)	No	Yes	No	No	-	No	-	**Critically Low**
Weaver 2013 (SR)	Partial Yes	Yes	Partial Yes	Yes	-	Yes	-	**High**

MA – meta-analysis; SR – systematic review

Critical domains: item 2 – protocol registered before commencement of the review; item 4 – adequacy of the literature search; item 7 – justyfication for excluding individual studies; item 9 – risk of bias from individual studies being included in the review; item 11 – appropriateness of meta-analytical methods; item 13 – consideration of risk of bias when interpreting the results of the review; item 15 – assessment of presence and likely impact of publication bias.
